# Editorial: Non-coding RNAs in ophthalmic diseases

**DOI:** 10.3389/fgene.2022.1055701

**Published:** 2022-11-10

**Authors:** Yedi Zhou, Songshan Li, Shigeo Yoshida

**Affiliations:** ^1^ Department of Ophthalmology, The Second Xiangya Hospital, Central South University, Changsha, Hunan, China; ^2^ Hunan Clinical Research Center of Ophthalmic Disease, Changsha, Hunan, China; ^3^ State Key Laboratory of Ophthalmology, Zhongshan Ophthalmic Center, Sun Yat-Sen University, Guangzhou, China; ^4^ Department of Ophthalmology, Kurume University School of Medicine, Kurume, Fukuoka, Japan

**Keywords:** non-coding RNA, ophthalmic disease, microRNA, long non-coding RNA, circular RNA, tRNA-related fragments

With the rapid development of high-throughput sequencing technology and bioinformatics, a growing number of studies revealed the key roles played by non-coding RNAs in the pathogenesis of ophthalmic diseases. In the current Research Topic, the authors discussed the roles and mechanisms of a variety of non-coding RNAs, including microRNAs (miRNAs), long non-coding RNAs (lncRNAs), circular RNAs (circRNAs), and tRNA-related fragments (tRFs). A range of ophthalmic diseases was discussed in this Research Topic such as proliferative diabetic retinopathy (PDR), retinoblastoma (RB), vernal keratoconjunctivitis (VKC), uveitis, and thyroid-associated ophthalmopathy (TAO).

Through high-throughput sequencing, Li et al. identified differentially expressed exosomal circRNAs, miRNAs, and mRNAs in the serum of PDR patients, in particular, they also proved the pro-angiogenic capability of circFndc3b derived from high-glucose-induced endothelial cells. In a brief research report, Syed et al. identified a total of 51 dysregulated miRNAs in the tears of children with VKC compared with controls. Through target gene prediction of these miRNAs, they also predict the probable involvement of the NF-kappa pathway, the cytokine signaling pathway and Treg cells in the VKC inflammatory regulations. Li et al. conducted a transcriptome analysis using the iris tissues of rats with experimental autoimmune uveitis compared with the control rats. They focused on lncRNA and mRNA expression changes, and recognized that the NOD-like receptor signaling pathway might be involved in the pathogenesis of autoimmune uveitis *via* bioinformatics analysis. Another article of systemic review by Tang et al. also focused on uveitis, in this study, they summarized the regulatory roles of miRNAs in Th17/Treg homeostasis, which is important in the pathogenesis of autoimmune uveitis. The article by Yue et al. identified the differential expressions of tRFs, a novel type of small non-coding RNAs, in patients with TAO, and also predicted the target genes and their possible functions and involved pathways by bioinformatics analysis.

A review article by Fernandez-Diaz et al. summarized the role played by lncRNAs, circRNAs and miRNAs in the physiopathological process of RB, and these non-coding RNAs can also be considered for application as novel potential biomarkers and therapeutic targets of RB. The review article by Rad et al. described the application potential and advantages of mesenchymal stem cell-derived exosomes along with their ingredients including miRNAs in the therapies of ophthalmic diseases.

Overall, the Research Topic shed light on the importance of non-coding RNAs in a variety of pathogenesis in ophthalmic diseases. These original and review articles indicated that non-coding RNAs, including miRNAs, lncRNAs, circRNAs, and tRFs, may play important roles in regulating cellular processes including gene regulation, pro-angiogenesis, and inflammation, and contribute to the initiation and development of ophthalmic diseases. Further studies should be carried out to deeply understand the specific roles and mechanisms of the dysregulated non-coding RNAs in these ocular diseases. To clarify the molecular regulatory mechanisms, it is also suggested to combine transcriptomics with genomics, proteomics, and metabolomics for multi-omics analysis.

